# Importance of the interferon-α system in murine large intestine indicated by microarray analysis of commensal bacteria-induced immunological changes

**DOI:** 10.1186/1471-2164-9-192

**Published:** 2008-04-26

**Authors:** Kaori Munakata, Masahiro Yamamoto, Naoko Anjiki, Mitsue Nishiyama, Sachiko Imamura, Seiichi Iizuka, Kiyoe Takashima, Atsushi Ishige, Kyoji Hioki, Yasuyuki Ohnishi, Kenji Watanabe

**Affiliations:** 1 Center for Kampo Medicine, School of Medicine, Keio University, 35 Shinano-machi, Shinjuku-ku, Tokyo 160-8582, Japan; 2 Pharmacology Research Department, Tsumura Central Research Laboratories, Tsumura & Co., 3586 Yoshiwara, Ami-machi, Inashiki-gun, Ibaraki 300-1192, Japan; 3 Graduate School of Natural Science and Technology, Kanazawa University, Kakuma-machi, Kanazawa 920-1192, Japan; 4 Central Institute for Experimental Animals, 1430 Nogawa, Miyamae-ku, Kawasaki, Kanagawa 216-0001, Japan

## Abstract

**Background:**

Although microbiota play a critical role in the normal development and function of host immune systems, the underlying mechanisms, especially those involved in the large intestine (LI), remain unknown. In the present study, we performed transcriptome analysis of the LI of germ-free (GF) and specific pathogen-free (SPF) mice of the IQI strain, an inbred strain established from ICR mice.

**Results:**

GeneChip analysis, quantitative real-time RT-PCR, and reconfirmation using bacteria-inoculated GF mice revealed differences in the expression levels of several immune-related genes, such as cryptdin-related sequences (CRS), certain subsets of type 1 interferon (IFN)-related genes, class Ib MHC molecules, and certain complements. LI expressed no authentic cryptdins but predominantly expressed CRS2, 4, and 7. The mRNA levels of IFN-related genes, including Irf7, Isgf3g, Ifit1 and Stat1, were lower in SPF- and flora-reconstituted mice. When an oral IFN-α inducer tilorone analog, R11567DA, was administered to SPF mice, IFN-α was induced rapidly in the LI at 4 h, whereas no IFN-α protein was detected in the small intestine (SI) or blood. In situ hybridization and immunohistochemistry suggested that the IFN-α production originated from Paneth cells in the SI, and portions of lamina proprial CD11b- or mPDCA1-positive cells in the LI.

**Conclusion:**

The present study suggests that microbial colonization, while inducing the expression of anti-microbial peptides, results in the down-regulation of certain genes responsible for immune responses, especially for type I IFN synthesis. This may reflect the adaptation process of the immune system in the LI to prevent excessive inflammation with respect to continuous microbial exposure. Further, the repertoire of anti-microbial peptides and the extraordinary role of interferon producing cells in the LI have been found to be distinct from those in the SI.

## Background

Humans harbor an enormous number of microbes in the intestine. Intestinal flora have been suggested to play a critical role in the normal development and physiology of host animals. Symbiosis of human commensal bacteria forms a metasystem of nutrient uptake and endobiotic/xenobiotic metabolism whose capacities greatly exceed those provided by the products of genes encoded in the human genome [1]. Further, gut microbiota are a key regulator of the human immune system. The immune response must be balanced between defending against pathogens while at the same time recognizing commensals as harmless [1-3]. The immune system of the small intestine, which is comprised of a variety of regulatory and effector compartments including Peyer's Patch cells, intraepithelial lymphocytes, lamina propria mononuclear cells and intestinal epithelial cells, has been extensively documented [4,5]. The normal colon, however, apparently lacks important structures/components such as Peyer's patches (PP) and Paneth cells, and little is known about the immunology of the large intestine, despite the marked differences in both function and luminal environment between the different regions of the intestine [6,7].

Previous studies using germ-free (GF)-, bacteria-reconstituted GF-, specific pathogen-free (SPF)- and conventional (CV)- mice have revealed that enteric bacteria have profound effects on the number and population of immune cells in PP [4,8,9], differentiation of Paneth cells [10-12], migration of T cells bearing α/β T cell receptor into the intestinal epithelium [13], development of IFN-γ-driven immune function [14,15] such as Th1 skewing [16] and MHC class II antigen expression [17,18], and oral tolerance induction [8,16,19]. Although functional genomics have demonstrated that enteric bacteria affect the expression of genes involved in the mucosal barrier and immunological defense [20,21], the mechanisms by which these immunological changes are induced, especially in the large intestine, are still obscure.

In the present study, transcriptome analysis was performed on colon tissues from IQI mice, an inbred strain established from ICR mice [22]. IQI mice are an autoimmune-prone strain: they induce a high level of antinuclear auto-antibody following mercuric chloride treatment [23], have thymic B cells [24], show age-related development of Sjogren's syndrome-like sialadenitis [25], and exhibit spontaneous skin lesions in aged females [26]. Comparisons of picryl chloride-induced contact dermatitis in BALB/c and IQI mice suggested that enhanced antigen presentation capacity in the ear dermis of IQI mice resulted in prominent T cell infiltration and more severe dermatitis as compared with BALB/c mice [26,27]. High sensitivity in the mucosal sites in IQI mice suggests that this strain may be useful for investigating changes in mucosa-associated immune systems induced by bacterial burden. The present results demonstrated that bacterial colonization increases the expression of cryptdin-like products and decreases the expression of regulatory factors that are indispensable prerequisites for massive IFN-α synthesis. Our investigation of the cells responsible for these changes by in situ hybridization and time course analysis of IFN-α production suggested that IFN-α plays an important role in the defense response in the large intestine.

## Results

### GeneChip analysis of the large intestines of GF and SPF mice

There were 11 probe sets whose expression levels in SPF mice were significantly (> 2 fold) higher than in GF mice (Table 1). Among these 11 probe sets, 6 were those of the cryptdin family. When we applied the same criteria, there were 43 probe sets expressed at lower levels in SPF mice than in GF mice (Table 2). The list of decreased genes contains 11 genes whose expression has been reported to be induced by type-1 interferon. Table 1 lists glycoprotein galactosyltransferase-alpha 1,3 (Ggta1) and Table 2 lists Ifit1/GARG16, and four and a half LIM domains 1 (Fhl1), which have been noted to change in SPF mice or colonized GF mice in previous reports [20,28]. However, there are only a few additional genes, whose function is considered to be "professionally" immune-related.

**Table 1:** Genes more highly expressed in large intestines of SPF mice than those in GF mice

Probe Set ID	Gene Name*	Gene Symbol	Fold Change**	p-value
160918_at	S100 calcium binding protein G	S100 g	10.16	0.006
100351_f_at	**defensin related cryptdin 3/defensin-related cryptdin 23**	Defcr3/Defcr23	5.27	0.049
99551_f_at	**defensin related cryptdin 5**	Defcr5	4.79	0.022
93863_f_at	**defensin related cryptdin 3/defensin related cryptdin 6/defensin-related cryptdin 23/defensin-related cryptdin 24**	Defcr3/Defcr6/Defcr23/Defcr24	4.30	0.034
93879_f_at	**defensin related cryptdin 3**	Defcr3	4.00	0.031
92812_f_at	**defensin related cryptdin 3/defensin related cryptdin 4/defensin related cryptdin 6/RIKEN cDNA 2010016F14 gene/defensin-related cryptdin 23/defensin-related cryptdin 24**	Defcr3/Defcr4/Defcr6/2010016F14Rik/Defcr23/Defcr24	3.67	0.018
160909_at	small proline-rich protein 1A	Sprr1a	3.64	0.046
100884_at	aldo-keto reductase family 1, member B8	Akr1b8	2.89	0.001
102993_at	glycoprotein galactosyltransferase alpha 1, 3	Ggta1	2.88	0.016
101794_f_at	**defensin-related cryptdin 23**	Defcr23	2.56	0.034
93755_at	resistin like beta	Retnlb	2.00	0.015

*The genes of defensin family are shown in bold font.

**Fold change is defined as the ratio of the signal of SPF to that of GF.

**Table 2:** Genes more highly expressed in large intestines of GF mice than those in SPF mice

Probe Set ID	Gene Name	Gene Symbol	Fold change*	p-value
**Type 1 interferon-related genes**				
104750_at	interferon gamma inducible protein 47	Ifi47	5.13	0.003
103639_at	interferon-induced protein with tetratricopeptide repeats 2	Ifit2	4.87	0.009
102906_at	T-cell specific GTPase	Tgtp	3.99	0.036
100981_at	interferon-induced protein with tetratricopeptide repeats 1	Ifit1	3.81	0.013
103202_at	guanylate nucleotide binding protein 4	Gbp4	3.76	0.030
104177_at	radical S-adenosyl methionine domain containing 2	Rsad2	3.20	0.006
97409_at	immunity-related GTPase family, M	Irgm	3.03	0.008
101465_at	signal transducer and activator of transcription 1	Stat1	2.63	0.025
104669_at	interferon regulatory factor 7	Irf7	2.58	0.022
103335_at	lectin, galactose binding, soluble 9	Lgals9	2.26	0.042
104597_at	guanylate nucleotide binding protein 2	Gbp2	2.15	0.035
**Immunoglobulins**				
96969_at	similar to Ig V-K167 precursor	LOC381776	7.39	0.014
93904_f_at	similar to Ig H-chain (VDJ-region) precursor	LOC238418	6.11	0.023
101320_f_at	similar to IgM(b) heavy pre-chain (AA -18 to 119)/similar to anti-poly(dC) monoclonal antibody heavy chain/similar to immunoglobulin heavy chain/similar to Ig heavy chain V region 3 precursor/similar to monoclonal antibody heavy chain	V165-D-J-C mu etc	3.87	0.006
93584_at	immunoglobulin heavy chain 6 (heavy chain of IgM)/Unknown (protein for MGC:60843)	Igh-6/MGC60843	2.57	0.041
97575_f_at	Immunoglobulin heavy chain 1a (serum IgG2a), mRNA (cDNA clone MGC:6529 IMAGE:2651493)	Igh-VJ558	2.33	0.011
93638_s_at	immunoglobulin lambda chain, variable 1	Igl-V1	2.25	0.017
**Other molecules**				
103957_at	transferrin receptor	Tfrc	6.84	0.001
160986_r_at	angiotensin I converting enzyme (peptidyl-dipeptidase A) 2	Ace2	5.13	0.040
92699_at	solute carrier family 7 (cationic amino acid transporter, y+ system), member 9	Slc7a9	4.45	0.014
96792_at	apolipoprotein B	Apob	3.60	0.022
160933_at	interferon gamma induced GTPase	Igtp	3.39	0.003
94936_at	meprin 1 beta	Mep1b	3.33	0.011
96912_s_at	cytotoxic T lymphocyte-associated protein 2 alpha/cytotoxic T lymphocyte-associated protein 2 beta	Ctla2a/Ctla2b	3.30	0.000
102373_at	glutamyl aminopeptidase	Enpep	3.13	0.008
100030_at	uridine phosphorylase 1	Upp1	3.10	0.006
98994_at	solute carrier family 34 (sodium phosphate), member 2	Slc34a2	3.05	0.012
98410_at	interferon inducible GTPase 2	Iigp2	2.91	0.007
92689_at	interleukin 18 binding protein	Il18bp	2.82	0.021
103025_at	Moloney leukemia virus 10	Mov10	2.79	0.035
97950_at	xanthine dehydrogenase	Xdh	2.74	0.016
102559_at	bone morphogenetic protein 2	Bmp2	2.49	0.020
96764_at	interferon inducible GTPase 1	Iigp1	2.48	0.001
94088_at	polypyrimidine tract binding protein 2	Ptbp2	2.42	0.012
97500_g_at	four and a half LIM domains 1	Fhl1	2.32	0.049
98958_at	enhancer of yellow 2 homolog (Drosophila)	Eny2	2.30	0.021
103250_at	deafness, autosomal dominant 5 homolog (human)	Dfna5h	2.26	0.042
97994_at	transcription factor 7, T-cell specific	Tcf7	2.25	0.026
92877_at	transforming growth factor, beta induced	Tgfbi	2.23	0.023
102965_at	expressed sequence AI481105	AI481105	2.17	0.019
104606_at	CD52 antigen	Cd52	2.15	0.004
99463_at	cytochrome P450, family 3, subfamily a, polypeptide 13	Cyp3a13	2.14	0.011
99993_at	alanyl (membrane) aminopeptidase	Anpep	2.08	0.017

* Fold change is defined as the ratio of the signal of GF to that of SPF.

### Verification of the changes in gene expression by RT-PCR

We next attempted to confirm the GeneChip data by using quantitative real time RT-PCR. Because the cryptdin family consists of more than 20 genes with DNA sequences of extraordinarily high homology, we determined the gene identity by DNA sequencing. The RT-PCR fragments amplified using the primers common for the cryptdin family were cloned into pT7Blue vector. DNA sequencing has suggested that the members of cryptdin family predominantly expressed in the colon of IQI GF and SPF mice are all cryptdin-related sequences; i.e. Defcr-rs 4, 2, and 7 (40, 7, 10 of GF mice-derived 62 clones and 24, 15, 16 of SPF-mice derived 61 clones, respectively). No authentic cryptdins were found among the sequences of the aforementioned 123 clones. Because of the difficulties in designing the primer/probe sets, due to extremely high sequence homology among these genes, further investigation using quantitative RT-PCR has not been done.

When we extended the criteria to genes with a greater than 1.5-fold ratio of expression in GF mice as compared with SPF mice, an additional class of immune-related genes were identified; namely, complement-related genes and MHC-related genes. The latter contains non-classical MHC class 1b genes which are known to be monomorphic or oligomorphic and expressed in a limited number of tissues, such as thymus and intestinal epithelium [29,30]. Furthermore, MHC I genes are known to be type 1 IFN-inducible genes whose promoter regions contain typical interferon stimulus response elements (ISREs) [31,32]. In addition to the immune-related genes listed in Table 2, additional genes (for example, several interferon regulatory factors, IFN-α-inducible genes and IFN receptors) were assessed by RT-PCR analysis (Table 3) to obtain a more detailed picture of the changes in type 1 IFN-related molecules. The expression of IFN-α s (α1, α2, α4), the end products of these signaling cascades, has also been analyzed but expression of IFN-αs was not detected in either SPF or GF mice (data not shown).

**Table 3:** Verification of the changes in gene expression by quantitative realtime RT-PCR

Gene Name*	Gene Symbol	qt realtime RT-PCR		GeneChip	RT-PCR primer/probe ID***
				
		Fold change**	p value	significant difference	GeneChip Probe Set ID
**Type 1 interferon-regulatory factors**					
interferon regulatory factor 1 (IRF1)	Irf1	1.00	-	-	Mm00515191_m1 102401_at
interferon regulatory factor 3 (IRF3)	Irf3	1.06	0.479	-	Mm00516779_m1 99103_at
interferon regulatory factor 5 (IRF5)	Irf5	2.38	0.112	-	Mm00496477_m1 93425_at
interferon regulatory factor 6 (IRF6)	Irf6	1.50	0.363	-	Mm00516797_m1 92440_at
**interferon regulatory factor 7 (IRF7)**	Irf7	2.44	0.031	+	Mm00516788_m1 104669_at
**interferon dependent positive acting transcription factor 3 gamma(IRF9)**	Isgf3g	2.53	0.001	-	Mm00492679_m1 103634_at
interferon consensus sequence binding protein 1(IRF8)	Irf8	1.11	0.299	-	Mm00492567_m1 98002_at
**Type 1 interferon-inducible genes**					
**interferon-induced protein with tetratricopeptide repeats 1**	Ifit1	5.22	0.048	+	Mm00515153_m1 100981_at
interferon-induced protein with tetratricopeptide repeats 2	Ifit2	0.82	0.861	+	Mm00492606_m1 103639_at
glucocorticoid-attenuated response gene 49 (GARG-49/IRG2)	Ifit3	1.99	0.195	-	Mm01704846_s1 93956_at
guanylate nucleotide binding protein 2:mGBP-2	Gbp2	2.83	0.091	+	Mm00494575_m1 104597_at
expressed sequence AI481100:GTPI	Iigp2	2.11	0.060	+	Mm00546343_s1 96764_at
chemokine (C-X-C motif) ligand 10:IP-10	Cxcl10	4.69	0.058	-	Mm00445235_m1 93858_at
interferon inducible protein 1:LRG-47, Ifi1	Irgm	6.30	0.188	+	Mm00492596_m1 97409_at
**lectin, galactose binding, soluble 9**	Lgals9	1.96	0.023	+	Mm00495295_m1 103335_at
**interferon gamma induced GTPase**	Igtp	2.95	0.012	+	Mm00497611_m1 160933_at
**Type I interferon receptors**					
interferon (alpha and beta) receptor 1	Ifnar1	0.29	0.424	-	Mm00439544_m1 100483_at
interferon (alpha and beta) receptor 2	Ifnar2	1.12	0.599	+	Mm00494916_m1 101014_at
**Complement-related genes**					
complement component 1, q subcomponent, alpha polypeptide	C1qa	2.24	0.105	+	Mm00432142_m1 98562_at
complement component 3	C3	2.74	0.059	+	Mm00437858_m1 93497_at
serine (or cysteine) proteinase inhibitor, clade G, member 1:complement 1 inhibitor	Serping1	1.41	0.070	+	Mm00437834_m1 99081_at
**MHC-related genes**					
Histocompatibility 2, class II antigen A, alpha	H2-Aa	1.73	0.197	+	Mm01609331_m1 92866_at
**MHC class II H2-I-A-beta gene (k haplotype)**	H2-AB1	1.82	0.022	+	H2-AB1-TG04 100998_at
MHC (A.CA/J(H-2K-f) class I antigen	H2-Q1	0.88	0.496	+	Mm00657093_g1 97125_f_at
**MHC class I Q4 beta-2-microglobulin (Qb-1) gene**	H2-Q4	1.65	0.008	-	H2-Q4-TG09 99378_f_at
**Q8/9d gene**	LOC386462	2.22	0.017	+	H2-Q78-TG08 98438_f_at
Histocompatibility 2, T region locus 10+17	H2-T10/22/9	1.74	0.114	+	H2-T1017-TG11 93865_s_at
Histocompatibility 2, T region locus 23	H2-T23	1.35	0.116	+	Mm00439246_g1 98472_at
**Other molecules**					
**cytotoxic T lymphocyte-associated protein 2 alpha**	Ctla2a	5.17	0.002	+	Mm00484032_g1 96912_s_at
**CD52 antigen:CD80?**	Cd52	2.40	0.029	+	Mm00489055_m1 104606_at

* The genes giving a significant (p < 0.05) result in the real-time RT-PCR were shown in bold font.

** Fold change is defined as the ratio of the signal of GF to that of SPF.

*** The primer probe sets were perchased from TaqMan Gene Expression Assays (Applied Biosystems) except for those for H2-AB1, H2-Q4, LOC386462, and H2-T10/22/9, which were synthesized by Costum TaqMan gene expression assay (Appried Byosystems) using File Builder program (Applied Byosystems). The primer-probe IDs in TaqMan Gene Expression Assays were shown in the table. The sequences of the primer-probe sets in Custom TaqMan gene expression assay are the following.

**H2-AB1-TG04 **forward; 5'-CCTGAAGAGCCCCATCACT-3', reverse; 5'-CTTGCTCCGGGCAGACT-3', probe 5'-ACTGTGCCCTCCACTCCA-3'

**H2-Q4-TG09 **forward;5'-CCTGGAGCTCGGGAAGGA-3', reverse; 5'-CCGTCAGATCTGTGGTGACATG-3', probe; 5'-ACAGATCCTCCAAAGGCA-3'

**H2-Q78-TG08 **forward; 5'-AGGGCCATGAGCAGAGTTTC-3', reverse; 5'-CCCCATGTCACAGCCATACA-3', probe; 5'-AAGGGCGGCTCTCACA 3'

**H2-T1017-TG11 **forward; 5'-ACGTGCCATGTGGACCAT-3', reverse 5'-GCAACAATCCAAATCCAAGGCTTTT-3', probe; 5'-CAGGCTCCCATCTCAG-3'

### Verification study using microflora-reconstituted (ex-GF) mice

To verify whether the observed changes were attributable to the presence of intestinal flora, we examined gene expression in microbiota-reconstituted mice. Pregnant GF mice were cohabited with SPF mice beginning 3 to 5 days before delivery. The offspring was kept under SPF conditions until the age of 9 weeks and total RNA was prepared from the large intestine. Real time RT-PCR was performed, the results of which are shown in Table 4. Significant changes in gene expression were generally similar to those presented in Table 3.

**Table 4:** Changes in gene expression of flora-reconstituted mice (quantitative realtime RT-PCR)

Gene Name*	Gene Symbol	qt realtime RT-PCR		RT-PCR
			
		Fold change**	p value	primer/probe ID***
**Type 1 interferon-reguratory factors**				
interferon regulatory factor 1 (IRF1)	Irf1	1.00	-	Mm00515191_m1
**interferon regulatory factor 3 (IRF3)**	Irf3	1.84	0.003	Mm00516779_m1
interferon regulatory factor 5 (IRF5)	Irf5	3.05	0.053	Mm00496477_m1
interferon regulatory factor 6 (IRF6)	Irf6	6.47	0.055	Mm00516797_m1
**interferon regulatory factor 7 (IRF7)**	Irf7	4.42	<0.001	Mm00516788_m1
**interferon dependent positive acting transcription factor 3 gamma(IRF9)**	Isgf3g	2.87	0.033	Mm00492679_m1
interferon consensus sequence binding protein 1(IRF8)	Irf8	1.89	0.043	Mm00492567_m1
**Type 1 interferon-inducible genes**				
**interferon-induced protein with tetratricopeptide repeats 1**	Ifit1	4.13	0.029	Mm00515153_m1
interferon-induced protein with tetratricopeptide repeats 2	Ifit2	5.18	0.233	Mm00492606_m1
glucocorticoid-attenuated response gene 49 (GARG-49/IRG2)	Ifit3	12.77	0.095	Mm01704846_s1
guanylate nucleotide binding protein 2:mGBP-2	Gbp2	3.66	0.069	Mm00494575_m1
**expressed sequence AI481100:GTPI**	Iigp2	4.43	0.008	Mm00546343_s1
chemokine (C-X-C motif) ligand 10:IP-10	Cxcl10	14.33	0.133	Mm00445235_m1
interferon inducible protein 1:LRG-47, Ifi1	Irgm	7.14	0.177	Mm00492596_m1
**lectin, galactose binding, soluble 9**	Lgals9	4.03	0.008	Mm00495295_m1
**interferon gamma induced GTPase**	Igtp	3.22	0.132	Mm00497611_m1
**IFN- type I-induced and dsRNA-activated kinase**	Eif2ak2	2.07	0.040	Mm00440966_m1
**2'-5' oligoadenylate synthetase 1A**	Oas1a	3.18	0.046	Mm00836412_m1
2'-5' oligoadenylate synthetase 1B	Oas1b	13.05	0.052	Mm00449297_m1
**TAP binding protein**	Tapbp	1.86	0.046	Mm00493417_m1
**Type I interferon receptors**				
**interferon (alpha and beta) receptor 1**	Ifnar1	1.84	0.017	Mm00439544_m1
**interferon (alpha and beta) receptor 2**	Ifnar2	2.06	0.010	Mm00494916_m1
**Complement-related genes**				
complement component 1, q subcomponent, alpha polypeptide	C1qa	1.87	0.178	Mm00432142_m1
complement component 3	C3	2.08	0.141	Mm00437858_m1
**serine (or cysteine) proteinase inhibitor, clade G, member 1: complement 1 inhibitor**	Serping1	2.18	0.005	Mm00437834_m1
**MHC-related genes**				
Histocompatibility 2, class II antigen A, alpha	H2-Aa	2.72	0.266	Mm01609331_m1
**MHC class II H2-I-A-beta gene (k haplotype)**	H2-AB1	3.42	0.041	H2-AB1-TG04
MHC (A.CA/J(H-2K-f) class I antigen	H2-Q1	3.47	0.356	Mm00657093_g1
**MHC class I Q4 beta-2-microglobulin (Qb-1) gene**	H2-Q4	3.83	0.032	H2-Q4-TG09
Q8/9d gene	LOC386462	11.69	0.153	H2-Q78-TG08
**Histocompatibility 2, T region locus 10+17**	H2-T10/22/9	8.06	0.006	H2-T1017-TG11
Histocompatibility 2, T region locus 23	H2-T23	1.10	0.762	Mm00439246_g1
**Other molecules**				
cytotoxic T lymphocyte-associated protein 2 alpha	Ctla2a	9.48	0.139	Mm00484032_g1
CD52 antigen	Cd52	3.05	0.095	Mm00489055_m1
**signal transducer and activator of transcription 1**	Stat1	4.66	0.026	Mm00439518_m1
**signal transducer and activator of transcription 2**	Stat2	3.81	0.015	Mm00490880_m1

* The genes giving a significant (p < 0.05) result in the real-time RT-PCR were shown in bold font.

** Fold change is defined as the ratio of the signal of GF to that of SPF.

*** See the legend of Table 3.

### Production of type 1 interferon in the small and large intestines

**Figure 1:**
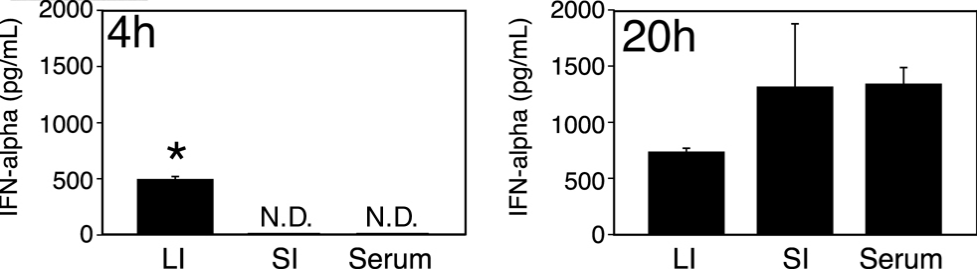
**The content of IFN-αs after R11567DA administration.** Four hours after oral administration of R11567DA at 100 mg/kg, the small and large intestines were removed, homogenized, and centrifuged. The amounts of IFN-αs were measured by ELISA as described in Materials and Methods. Data represent mean ± SEM (n = 3). LI, large intestine; SI, small intestine, N.D., not detected (under detection limit), *, p < 0.0001 vs SI. Without R11567DA treatment, IFN-αs were not detected in any tissues or sera. This experiment was repeated twice with similar results.

To investigate whether IFN-related genes play a significant role in the immune defense response in the large intestine, we administered an oral interferon inducer, R11567DA, and used ELISA to determine the amount of IFN-α produced in the small intestine, large intestine, and blood. As shown in Figure 1, after 4 hours, high levels of IFN-α were detected in homogenates of large intestine but not in small intestine or serum. After 20 hours, IFN-α was also detected in the small intestine and serum, and at higher levels than in large intestine. These data suggest that the large intestine can produce IFN-α very rapidly, prior to the elevation of blood IFN-α levels, even before the commencement of IFN-α production in the small intestine, which contains an abundance of IFN-signaling molecule mRNAs and would likely encounter the oral IFN inducers earlier than would the large intestine.

### In situ hybridization

**Figure 2:**
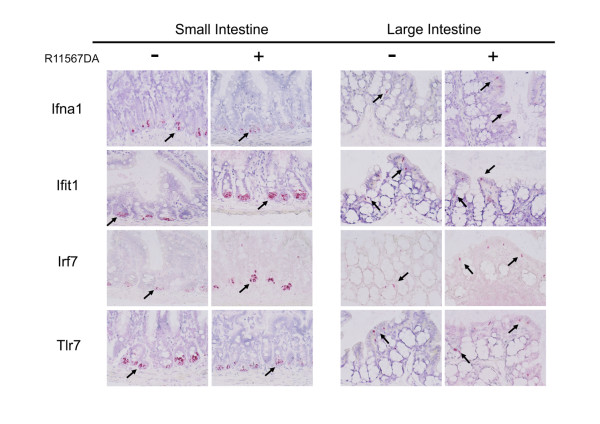
**In situ hybridization for mRNAs of IFN-α-related genes and Tlr7 in the small and large intestines.** A. Photomicrographs showing localization of Ifna1, Ifit1, Irf7 and Tlr7. In the small intestine, all signals for Ifna1, Ifit1, Irf7 and Tlr7 were detected in Paneth cells. In the large intestines, the signals were detected in the discrete cells distributed in lamina propria, though further examination is necessary to elucidate whether these signals were generated from the same cells, and to determine their cellular identity. Five sections per tissue were prepared from each of 8 mice. Representative photographs were shown.

**Figure 3:**
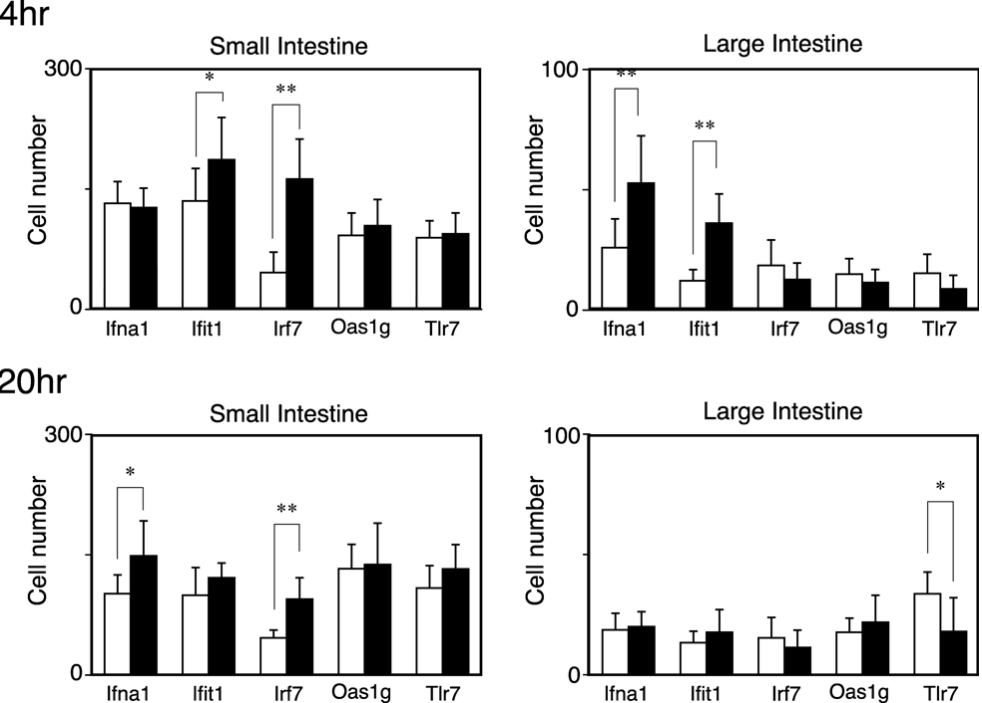
**Quantitation of the numbers of cells positive for Ifna1, Irf7, Ifit1, Oas1g, or Tlr7.** Open and closed columns represent data obtained from saline- and R11567DA- treated mice, respectively. Sections prepared from 8 mice per group were used. Five low-power photographs were taken for each section, and the number of positive cells was counted and normalized to per 0.8 mm^2 ^of intestinal mucosa. Data represent mean ± SEM. *P < 0.05, **P < 0.01, significantly different from the R11567DA (-) group.

To elucidate the cell types responsible for R11567DA-induced IFN-α production, we performed in situ hybridization on sections prepared from the small and large intestines with or without 4-hour R11567DA treatment in SPF mice (Figure 2 and Figure 3). In situ hybridization with an IFN-α1-specific probe revealed that IFN-α1 was expressed in Paneth cells, the small intestine, and discrete cells distributed in the lamina propria in the large intestine. Similar results have been obtained with probes for other types of IFN-α-induced genes, such as Ifit1, Irf7 (Figure 2) and oas1g (data not shown). The localization of Tlr7 mRNA, which has been shown to be expressed in a limited number of cell types, including plasmacytoid dendritic cells [33,34], showed a similar distribution pattern to that of the aforementioned IFN-related genes. Both at 4 and 20 hours, the number of IFN-α mRNA positive cells per mucosal area in the small intestine was larger than that in the large intestine (Figure 3). However, at 4 hours, R11567DA treatment increased the number of IFN-α-positive cells in the large intestine but not in the small intestine (Figure 3), which is in good accordance with the results of our IFN-α ELISA. The number of Ifit1-positive cells was increased by R11567DA treatment in both the small and large intestine, while the increase in the number of Irf7-positive cells occurred only in the small intestine. The number of Oas1g and Tlr7 did not change in either the small or large intestine following R11567DA treatment. At 20 hours, in the small intestines of R1156DA-treated mice, the number of IFN-α and Irf7-positive cells increased as compared with the control group, while no difference was seen in the large intestines. The number of Tlr7-positive cells decreased in the large intestine at 20 hour**s**.

### Immunohistochemistry

**Figure 4:**
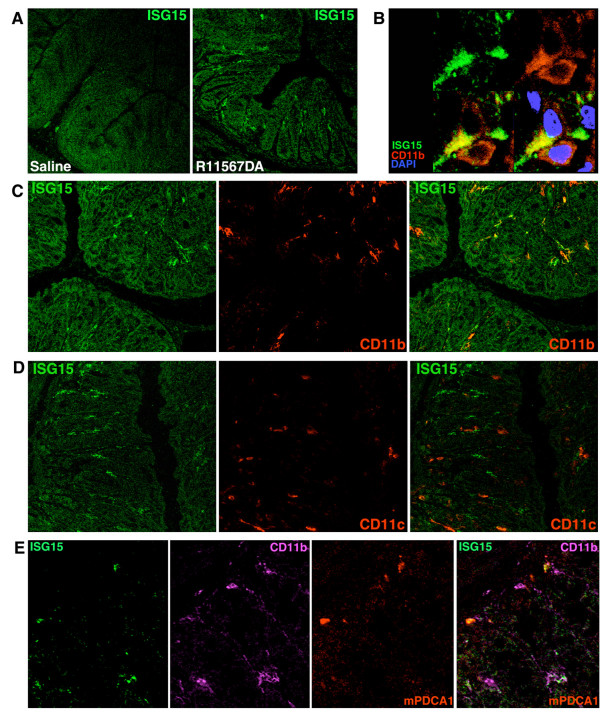
**Immunofluorescence detection of IFN-α producing cells.** Cryostat sections of colonic tissues from mice 4 hr after the administration of saline or R11567DA. Sections were multi-labeled with anti-ISG and anti-CD11b, anti-CD11c, anti-mPDCA1 or DAPI. A, ISG15 signals were observed in colonic lamina propria of R11567DA-treated mice while saline treatment produced no signal. B, C. Portions of ISG15^+ ^cells (green) were co-stained (yellow) with anti-CD11b antibody (red). Nuclei were visualized by DAPI staining (blue) in Panel B. D, ISG15^+^CD11c^+ ^double positive cells (yellow) were scarcely found. E, In addition to ISG15^+^CD11b^+ ^double-positive cells (yellow), ISG15^+^mPDCA1^+ ^double positive cells (white) were found. However, more than half of ISG15^+ ^cells was stained by neither anti-CD11b nor anti-mPDCA1 antibodies (data not shown).

To identify the cells responsible for R11567DA-induced IFN-α production, we performed immunohistochemistry on sections prepared from the large intestines with or without 4-hour R11567DA treatment in SPF mice. The antibody to ISG15, a typical type-1 interferon-stimulated gene stained discreet single cells or relatively small cell aggregates in the lamina propria in R11567DA-treated colons (Figure 4A). No signal was detected in the epithelial layers. Multiple staining by combinations of antibodies including dendritic cell markers revealed that in the large intestine ISG15 was produced in a small proportion of CD11b^+ ^cells (Figure 4B, C, E) and mPDCA1^+ ^cells (Figure 4E) although more than half of the ISG15^+ ^cells were not stained by either CD11b or mPDCA1 antibody. Virtually no ISG15^+ ^cells were found among the CD11c^+ ^cells (Figure 4D).

## Discussion

In the present study, transcriptome analysis was performed on colon tissue. This approach to profile transcription in whole tissues may be limited in that the detected changes are derived from composite changes in plural cell types. However, previous transcriptome studies that have focused on intestinal epithelial cells (IECs) [20,28] did not appear to adequately characterize the microflora-induced immunological changes in terms of transcriptional profiling, even in the small IECs, the physiology of which is supposed to be profoundly affected by a variety of GALT-derived cells and/or mediators. Although recent studies have revealed a wide array of immune-oriented functions of IECs [35,36], the spectrum of immune functions carried out by IECs is limited. By analyses of gene expression using whole colons from commensal GF mice, certain bacteria have been reported to increase the expression of IFN-γ-related genes and immunoglobulins [14,15,21].

In the present study, the expression of CRSs was increased by commensal bacteria. CRSs are cationic peptides that have a pro-region with high similarity to the pro-region of α-defensins. Although mature CRS peptides have no sequence homology or structural homology to any other known anti-microbial peptides, CRSs have been reported to co-localize with cryptdins in the granules of Paneth cells in similar amounts to cryptdins and to have potent microbial bacteriacidal activity [37-39]. A previous study using the differential display technique showed higher expression of CRS4C in the small intestines of SPF ICR mice than of GF ICR mice [40]. If CRSs function as the first-line defense against microbial invasion, like other various intestinal anti-microbial peptides, it is reasonable that the expression of CRSs in the small and large intestines of SPF mice is higher than that of GF mice. However, apparently normal colons do not have Paneth cells, which are the main sites of cryptdin and CRS localization in the small intestine. Although metaplastic Paneth cells expressing antimicrobial proteins such as cryptdins and lysozymes are known to appear in inflammed colons [41,42], histological examination detected no such cells in the large intestines of SPF mice (data not shown). Because cryptdins have been reported to also be present in the epithelial cells of the small intestines [40], CRSs may be expressed in the colonic epithelium. The localization and biological implication of CRSs remains to be elucidated in future research.

The most interesting finding of the present study is the possible importance of IFN-α in the defense systems of the large intestine. The most prominent difference in the expression of professional immune molecules was observed for genes involved in the IFN-α induction pathway. Many of them, such as Ifit1, 2 [43], Ifi47 [44], Tgtp [45], Gbp2 and 4 [46], Irgm [47]. Lgals9 [48], and Rsad2 [49] were originally identified as IFN-α-inducible genes. One molecule, Irf7, is a rate limiting transcription factor located in the center of a self-amplifying positive feedback loop of massive IFN-α production [50]. Microbial infection induces phosphorylation of IRF7 protein, and phosophorylated IRF7 is transported into nuclei to strongly induce the expression of IFN-αs and Irf7 itself. In the presence of kinase activated by infectious agents, newly synthesized IRF7 continues to activate the loop resulting in the explosive production of IFN-αs. The inducibility of IFN-α production in various tissues/cells and their steady state level of IRF7 proteins are known to be closely correlated [51]. Isgf3g (Irf9) and Stat1 are 2 components of ISGF3, a signaling complex that transduces the signal from receptors of IFN-α/β and induces the expression of IFN-α-related molecules including double-stranded RNA-dependent kinase Eif2ak2 (Prkr) [52], 2'-5' oligozdenylate synthetase 1A (Oas1a or Oas1g) [53], ISG15 ubiquitin-like modifier (G1p2 or Isg15) [54], and especially, Stat1 and Irf7 [55]. Changes in the expression of Stat2, the other component of ISGF3, have also been observed. In contrast, the expression of other interferon regulatory factors such as Irf1, Irf2, and Irf8 did not show differences, and expression of end-products such as the IFN-αs, a2 and a4 was not detected. These data suggest that, although GF and SPF mice usually do not express IFN-α, or do so at extremely low levels, once the stimulatory signals are triggered, they might produce different levels of IFN-α due to the difference in basal expression levels of rate-limiting regulatory factors of IFN-α production.

It is unknown, among the wide array of signaling molecules involved in IFN-α production, why only the relatively confined members showed differences in the present study. One possible explanation was provided by the results of IFN-α induction experiments. Administration of oral IFN-α inducers resulted in elevated tissue IFN-α content in both the small and large intestines. However, in spite of the high basal levels of IFN-α-related genes, including Irf7, the production of IFN-α in the small intestine occurred later than in the large intestines. The extreme rapidness of IFN production in the large intestine suggests a possible involvement of Type 1 interferon producing cells (IPCs), which are virtually identical to plasmacytoid dendritic cells (pDCs) [56]. IPCs express extremely high levels of Irf7 mRNA and protein constitutively, and can produce 100 to 1000 times more IFN than other blood cell types within several hours following stimulation.

This assumption was further supported by our in situ hybridization analysis using specific probes against the IFN-α-inducible genes, Ifnal, Ifit, Irf7, and Oas1g, and the plasmacytoid dendritic cell marker Tlr7. In the small intestine, histological analysis clearly indicated that the cells which had positive signals for type 1 IFN-related genes and Tlr7 were mainly Paneth cells. In the large intestine, all signals were located in the mononuclear cells scattered predominantly in the lamina propria, although further examination is necessary to determine whether these signals were generated from the same cells. In accordance with the results of our IFN-α ELISA, the number of Ifna1-positive cells increased in the large intestine but not in the small intestine at 4 hours. The increase in Irf7-positive cells in the small intestine may reflect amplification of the IRF7 pool, which is a prerequisite for massive IFN production in most cells types except for IPCs. In good agreement with the results of IFN-α ELISA at 20 hours, the numbers of IFN-α- and Irf7-positive cells increased in the small intestine. The increase in the number of these cells in the large intestine ceased at this time point, suggesting that the detected immunoreactive IFN-α might represent circulating IFN-α in the blood.

These data, collectively, address the possibility that IFN production and biological defense by IFN in the large intestine is borne mainly by IPCs, presumably recruited from the bloodstream to the intestinal lamina propria.

Finally, we performed immunohistochemical analysis to confirm whether the cells responsible for IFN-α production in the present study are IPCs/pDCs. We have screened several antibodies raised against type 1 IFN-related genes and found that the antibody for ISG15, a well-known IFN-stimulated gene [55,57], stained the cells in the lamina propria of the colon at 4 hours after IFN-α inducer treatment. Multiple staining by a combination of anti-ISG15 antibody and various CD markers has demonstrated that ISG15^+ ^cells contains CD11b^+ ^cells and mPDCA1^+ ^cells but not CD11c^+ ^cells. Conventionally, pDCs (or IPCs) have been identified as CD11c^+^B220^+^Gr-1^+ ^cells or mPDCA1^+ ^cells. However, recent studies suggested the phenotype of pDCs, especially in the peripheral tissues, may have a wide variation [58,59]. Takenaka S. et al [33] reported that, in the colons of BALB/c and C57BL/6 mice, many mPDCA1^+ ^cells exist but they are neither CD11c^+ ^nor CD11b^+^, and no typical CD11c^+^B220^+^Gr-1^+ ^pDCs were present. These findings have also been obtained in the colons of IQI mice in our study (data not shown): 1) CD11b^+ ^cells were predominant in the colonic lamina propria; 2) the majority of mPDCA1^+ ^cells was stained by neither anti-CD11b nor anti-CD11c antibodies; 3) typical pDCs (i.e., CD11c^+^B220^+^Gr-1^+ ^cells) were virtually absent. We have found that some CD11b^+ ^cells and mPDCA1^+ ^cells were co-stained with anti-ISG15 antibody, but more than half of ISG15^+ ^cells were double-negative for CD11b and mPDCA1. Colonic IPC may therefore be comprised of multiple cell populations with unique phenotypic characteristics distinct from conventional pDCs/IPCs. Accordingly, IFN-α production in IBD model mice has been noted in both CD11b^+ ^and CD11c^+ ^dendritic cells in colonic lamina propria [60]. However, the sensitivity and specificity of immunohistochemistry are limited and further extensive studies are necessary to clarify the functional and phenotypic characteristics of colonic IPCs.

Among the immune-related genes whose expression differed in the presence and absence of intestinal flora in the present GeneChip analysis, the IFN-related genes, MHC-related genes, some complements, and immunoglobulins were expressed at lower levels in SPF mice than in GF mice. This seems somewhat paradoxical because it is plausible that contact with high levels of microorganisms in SPF mice may result in enhancement of certain immunological defense systems in the large intestine. Along this line, Chowdhury et al. [61] reported that the expression of IFN-related genes including Irf7, Stat1, Stat2 and Tap1 in the small intestines was increased by the colonization of GF piglets with adult conventional swine feces. The discrepancy between our data and theirs may be explainable by the differences in various experimental settings such as the host animal (mouse vs. swine), RNA source (large intestine tissue vs. small intestinal epithelial cell), microbial status (SPF vs. conventional), and age of animals (9 vs. 2 weeks old). However, we think that the difference in the period after colonization may be responsible for the difference in expression of IFN-related genes between our study and theirs. The inflammation induced by microbial colonization of GF animals has been known to be only temporary and cease within several weeks [62,63]. After the inflammation is terminated, histological findings return to a state apparently indistinguishable from the normal intestines of mice genuinely harboring the microbes. Therefore, the up-regulation of IFN-related genes in the midst of or shortly after inflammation, and their down-regulation after a long period of resumption of integrity of tissues and adaptation to enteric microbes may be quite compatible and it appears that the IFN system may play an extraordinary role in the immunological confrontation, negotiation and reconciliation between microbiota and host animals. The immune systems of the gastrointestinal tract are known to have potent anti-inflammatory and regulatory properties. In the intestinal immune systems, constant exposure to bacteria-derived immunostimulating molecules may crowd up the threshold of activation of the inflammatory aspect of the immune system. Alternatively, the immune system may be programmed to develop anti-inflammatory and/or regulatory responses to microbial stimuli as a default setting, unless certain specialized machinery such as toll-like receptors provide the additional signature indicating the stimuli are derived from specific dangerous pathogens. The down-regulation of immune-related genes may represent certain acquired characteristics of the intestinal immune systems to adapt to circumstances in which immune cells are continually exposed to vast amounts of commensal bacteria.

## Conclusion

The present study suggests that microbial colonization, while inducing the expression of certain anti-microbial peptides, results in the down-regulation of a relatively limited range of genes responsible for host defense responses in the LI. This may reflect the adaptation process of immune systems in the LI to prevent excessive inflammation with respect to continuous microbial exposure. In particular, an extraordinary role of type I IFN has been suggested. Further, the repertoire of anti-microbial peptides and the extraordinary role of type 1 IFN-producing cells in the LI have been found to be distinct from those in the SI.

## Methods

### Animals

Male germ-free (GF) and specific pathogen-free (SPF) IQI/Jic mice were bred and maintained in the laboratory of the Central Institute for Experimental Animals (CIEA, Kawasaki, Kanagwa, Japan). GF mice were housed in a Trexler-type flexible film isolator in a standard germ-free state and screened on a weekly basis for germ-free status by sampling feces sterilely and culturing on MRS-agar plates under aerobic and anaerobic conditions. All GF-, SPF-, and ex-GF mice were kept on a 12:12-h light/dark cycle at a temperature of 22 ± 2°C. Microflora reconstitution was performed by transferring the pregnant females to an SPF facility, where they were reared in the presence of male SPF mice beginning at 3 to 5 days before the expected natal day, and the newborn male mice were used as microflora-reconstituted ex-GF mice. All animal procedures were approved by the institution's ethical committee for care and use of laboratory animals in research.

### Tissue dissection

Nine-week old mice were sacrificed by cervical dislocation and the colon was dissected (the colon was not divided into proximal and distal sections and was treated as a single intestinal section). Tissues destined for in situ hybridization were rinsed in phosphate-buffered saline (PBS), cut longitudinally, fixed in formalin or paraformaldehyde, and embedded in paraffin. Tissues destined for immunohistochemistry were embedded in OCT compound (Tissue-Tek, Sakura Finetechnical. Tokyo, Japan) and frozen in liquid nitrogen. Tissues destined for RNA extraction were flash-frozen in liquid nitrogen after washing with ice-cold PBS.

### Microarray analysis

Total RNA was extracted from mice (n = 3 per each group) using TRIzol (Life Technologies, Rockville, TX) and re-purified by RNeasy spin columns (Qiagen, Valencia, CA), according to the manufacturer's instructions. All samples were monitored using an Agilent Bioanalyzer (Agilent Biotechnologies, Boeblingen, Germany) and consistently demonstrated high-quality RNA (28S/18S ratio, ~2). The labeled cRNA prepared by in vitro transcription (Enzo Biochem, New York, NY) was fragmented, hybridized to an MG-U74Av2 array (Affymetrix, Santa Clara, CA) using an Affymetrix fluidics station, and scanned with an Affymetrix scanner, according to the Affymetrix protocols. Data were analyzed using the Affymetrix Microarray Suite (MAS) v.5.0 with all of the parameters set at default values (a global normalization was applied). The probe sets that had 2 or 3 absent A MAS detection calls per group (3 samples) in all groups were excluded; therefore, genes that had more than 2 present calls in any one of the groups were included in the analysis). Further, probe sets with signal intensities less than 50 were omitted because preliminary evaluation by RT-PCR revealed poor reproducibility for genes with lower signal intensities. Statistical analyses were performed by Welch's t-test. Affymetrix MAS5.0-normalized signal intensities were submitted to the Gene Expression Omnibus [64] and are available under the series ID: GSE8006.

### Real-time RT-PCR

All mouse primer and probe sets used TaqMan^® ^Gene Expression Assays (Applied Biosystems, Foster City, CA) or Custom TaqMan^® ^Gene Expression Assays (Applied Biosystems). Total RNA was purified from large intestine (n = 4–6 per group) as described in the previous paragraph. Reverse transcription was performed with 1 ug of total RNA using TaqMan Reverse Transcription Reagents (Applied Biosystems). Real time-PCR analysis was performed using an ABI Prism 7900HT (Applied Biosystems) with the following thermal cycling conditions: 1 cycle at 55°C for 10 min, followed by 40 cycles at 95°C for 15 sec and 60°C for 1 min. All samples were run in triplicates. Data were normalized by using Irf1, the expression of which had been found to be quite stable among groups in the present experimental setting, as determined by GeneChip and RT-PCR analyses. GADPH and beta-actin fluctuated greatly and slightly, respectively, which is presumably a reflection of the massive changes in tissue organization among microflora -absent and microflora -present intestines.

### Cloning and sequencing of CRSs

Fragments of the Defcrs family were amplified using the primers common for the family (forward, 5'-ATGAAGAGACTTGTCCTCCTC-3'; reverse, 5'-CTTCTTGAAGAGCAGAGCCTT-3') from total RNA from the large intestine of GF- and SPF mice by RT-PCR using TaqMan Reverse Transcription Reagents (Applied Biosystems) and Advantage HF2 PCR kit (Clonhech, Mountain View, CA). The fragments were cloned into pT7Blue vector using a pT7Tblue Perfectly Blunt Cloning Kit (Merck, Darmstadt, Germany) according to the manufacture's instructions. The inserts were checked by colony-direct PCR and sequenced using ABI PRISM^® ^3100 Genetic Analyzer (Applied Biosystems). The dendrogram was made from the sequence data by Contig Express and the Align X equipped in the Vector NTI advance Ver.9 (Invitrogen, Carlsbad, CA). BLAST searches on the NCBI database were performed to identify the gene name.

### Induction and quantitation of IFN-α protein

Tilorone analog R11567DA (Sigma, St.Louis, MO) was administered p.o. to IQI SPF mice at a dose of 100 mg/kg [65]. At 4 and 20 hours later, after cervical dislocation, blood samples were collected from the cardiac ventricle. The small and large intestines were then dissected and snap-frozen in liquid nitrogen. The blood was allowed to clot at room temperature for more than 30 minutes and centrifuged to obtain serum samples. The sera and intestines were stored at -80°C until use. At assay, the intestinal samples were thawed, homogenized in PBS buffer with protease inhibitor cocktail Mini (Roche Diagnotics, Basel, Switzerland), and centrifuged at 15000 g for 5 min at 4°C, and the supernatant was saved. The amount of IFN-α protein in the supernatant and serum was assessed using a commercially available IFN-α ELISA kit (PBL Biomedical Laboratories, Piscataway, NJ), which specifically detects the IFN-αs, α4, α5,α6 and α9.

### In situ hybridization

The protocol for in situ hybridization has been described previously [66]. Briefly, after acetylation, dehydration, and delipidation pretreatment, mRNAs in sections were hybridized with specific Brigati-Tail oligonucleotide probes using a MicroProbe Staining system (Falma, Tokyo Japan). The sequences of the probes used were Ifna1, 5'-TTCAGGGGAAATTCCTGCACCCCCA-3'; Ifit1, 5'-TTCGCAAAGCAGGCCATGGC-3'; Irf7, 5'-ATTTTCCGTGGCTGGGCCCACA-3'; Tlr7, 5'-TTTCCATGGTCCTGCTGGCCGA-3'; Oaslg, 5'-TTGGTTGGGCGCTGCTTCAGGA-3'. After post hybridization, the biotin-labeled hybrids were sequentially detected with alkaline phosphatase-conjugated streptavidin with stable DAB. Hybridization with antisense oligonucleotide probes for respective mRNAs gave no specific signals (data not shown).

### Immunohistochemistry

Immunohistochemistry was performed on frozen-sections of mid-colon post-fixed by acetone using the following primary antibodies, PE- or FITC-conjugated anti-CD11c (rat mAb, M1/70, BD Pharmingen, Franklin Lakes, NJ), PE- or FITC-anti-CD11b (hamster mAb, HL3, BD Pharmingen), PE- or FITC- or APC-anti-mPDCA1 (rat mAb, JF05-1C2.4.1, Miltenyi Biotec, Bergisch Gladbach, Germany), anti-ISG15 (rabbit pAb, AP1150a, Abgent, SanDiego, CA). Sections were incubated overnight at 4°C in primary antibodies diluted in antibody diluent (DAKO, Glostrup (Denmark), Secondary antibodies (Alexafluor-488-, or Alexafluor-647-labeled anti-rabbit IgGs) and DAPI (Molecular Probe), diluted in antibody diluent, were reacted for 2 h at room temperature. Sections were viewed by a laser-scanning confocal microscope FV1000 (Olimpus, Tokyo, Japan). In control sections, primary antibodies were omitted and no staining was observed in these sections.

## List of abbreviations

CRS: cryptdin-related sequences; CV: conventional; ex-GF: miroflora-reconstituted mice; GF: germ-free; IECs: intestinal epithelial cells, IFN: interferon; IPC: type 1 interferon producing cells; LI: large intestine; PP: Peyer's patches; SPF: specific pathogen-free.

## Authors' contributions

KM participated in most experiments. MY participated in the design of the study, immunohistochemistry, data analysis and coordination, and drafted the manuscript. NA carried out the anterior half of the experiments of this study. MN and KT participated in RT-PCR. SaI and SeI carried out in situ hybridization. KH and YO participated in the design and coordination of the microflora reconstitution experiments. AI participated in the design of the study and coordination. KW conceived the study and participated in its design and coordination. All authors read and approved the final manuscript.
